# Noncanonical NF-κB mediates the Suppressive Effect of Neutrophil Elastase on IL-8/CXCL8 by Inducing NKRF in Human Airway Smooth Muscle

**DOI:** 10.1038/srep44930

**Published:** 2017-03-21

**Authors:** Shu-Chuan Ho, Sheng-Ming Wu, Po-Hao Feng, Wen-Te Liu, Kuan-Yuan Chen, Hsiao-Chi Chuang, Yao-Fei Chan, Lu-Wei Kuo, Kang-Yun Lee

**Affiliations:** 1 School of Respiratory Therapy, College of Medicine, Taipei Medical University, Taipei, Taiwan; 2 Division of Pulmonary Medicine, Department of Internal Medicine, Shuang Ho Hospital, Taipei Medical University, New Taipei City, Taiwan; 3 Division of Pulmonary Medicine, Department of Internal Medicine, School of Medicine, College of Medicine, Taipei Medical University, Taipei, Taiwan; 4 Division of Pulmonary Oncology and Interventional Bronchoscopy, Department of Thoracic Medicine, Chang-Gung Memorial Hospital, Taoyuan, Taiwan; 5 Graduate Institute of Clinical Medicine, College of Medicine, Taipei Medical University, Taipei, Taiwan.

## Abstract

Neutrophil elastase (NE) suppresses IL-8/CXCL8 in human airway smooth muscle cells (hASM) while stimulating its production in respiratory epithelial cells. This differential effect is mediated by the selective induction of NKRF and dysregulation in chronic inflammatory diseases. We hypothesized that the differential activation of NF-κB subunits confer the opposite effect of NKRF on IL-8/CXCL8 in primary hASM and A549 cells stimulated with NE. The events occurring at the promoters of *NKRF* and *IL*-*8*/*CXCL8* were observed by ChIP assays, and the functional role of RelB was confirmed by knockdown and overexpression. Although p65 was stimulated in both cell types, RelB was only activated in NE-treated hASM, as confirmed by NF-κB DNA binding ELISA, Western blotting and confocal microscopy. Knockdown of RelB abolished the induction of NKRF and converted the suppression of IL-8/CXCL8 to stimulation. The forced expression of RelB induced NKRF production in hASM and A549 cells. NE activated the NIK/IKK1/RelB non-canonical NF-κB pathway in hASM but not in A549. The nuclear-translocated RelB was recruited to the *NKRF* promoter around the putative κB site, accompanied by p52 and RNA polymerase II. In conclusion, *NFRF* is a novel RelB-response gene, and NE is a stimulator of the non-canonical RelB/NF-κB pathway in hASM.

NF-κB-repressing factor (NKRF), previously known as NRF, is a constitutively expressed transcription factor that binds to the negative regulatory element (NRE) in the promoters of several NF-κB-responsive genes^1^. Originally designated as a “repressing factor”, NKRF actually plays a dual role depending on the cellular context. NKRF serves as a transcriptional coactivator in response to a few stimuli, e.g., IL-1 or different virus strains^1^,^2^,^3^. Nevertheless, in most of the other conditions, it represses NF-κB-driven transcription. In unstimulated cells, NKRF suppresses the basal transcription of the target genes, including IFN-γ, IL-8/CXCL8, iNOS, HIV type 1 long terminal repeat and IP-10^1^,^2^,^3^,^4^,^5^. Recently, the role of NKRF in human diseases has been unearthed. We first reported that the down-regulation of NKRF, partly due to oxidative stress, is linked to systemic inflammation in patients with chronic obstructive pulmonary disease (COPD)^6^. Following this, NKRF was demonstrated to be involved in irritable bowel syndrome with diarrhea (IBS-D)^7^ and to fine tune the cytokine response to active pulmonary tuberculosis^5^, as well as was correlated with the positive effect of allergen-specific immunotherapy^8^.

The regulation of NKRF is not completely understood. A few reports have focused on the post-transcriptional levels. Heterogeneous nuclear ribonucleoprotein D-like protein (JKTBP) 1 was demonstrated to be involved in the stabilization and IRES-dependent translation of NKRF mRNAs^9^. In addition, some microRNAs, including miR-301a^10^, miR-1290^11^ and miR-29^7^, were shown to target and reduce the expression of *NKRF* in pancreatic cancer, *Helicobacter pylori*-induced intestinal metaplasia of gastric epithelial cells and IBS-D, respectively. We have previously reported that NKRF is inducible at the transcriptional level in neutrophil elastase (NE)-stimulated hASM^12^. In this model, the inducible NKRF is bound to the promoter of *IL*-*8*/*CXCL8*, leading to the removal of RNA polymerase II and reduction of IL-8/CXCL8 mRNA expression in the presence of NF-κB. Intriguingly, NE does not induce NKRF in lung and bronchial epithelial cells such that it increases IL-8/CXCL8 expression. The molecular mechanisms whereby NKRF is sophisticatedly controlled in these two closely related structure cells are unclear.

Nuclear factor kappa B (NF-κB) is a ubiquitous transcription factor that regulates numerous genes controlling immune/inflammation, cell growth, apoptosis and tissue differentiation^13^,^14^,^15^. In mammalian cells, there are five NF-κB family members, e.g., RelA (p65), RelB, c-Rel, p50/p105 (NF-κB1) and p52/p100 (NF-κB2), which bind to the κB elements in the promoters and enhancers of responsive genes by forming hetero- or homo-dimers^16^,^17^,^18^. NF-κB activation can lead to gene transcription or repression by variable mechanisms, such as changing the component monomers and potential interactions with distinct transcriptional modulators^19^,^20^, thereby acting in a Janus-like manner^21^. In contrast to the well-known pro-inflammatory role of the p65/p50-mediated canonical NF-κB pathway^22^, a growing body of evidence has supported the hypothesis that the RelB-mediated non-canonical NF-κB pathway can suppress inflammation. RelB/p50 was initially found to inhibit TNF production in LPS-stimulated dendritic cells and macrophages^23^. Subsequently, RelB was demonstrated to mediate LPS tolerance in sepsis^24^,^25^ and confer a unique hypo-responsive phenotype of decidual endothelial cells to LPS^26^, possibly playing a role in the immune tolerance to microorganisms during pregnancy.

Analysis of the sequence of the *NKRF* promoter revealed a potential κB site (GGAGTTTCAC, −969 to −960 from the translation start site, NCBI CoreNucleotide: CH471161) with 80% similarity to the consensus κB site (GGGRNYYYCC where R is a purine, Y is a pyrimidine and N is any base)^27^. In this study, we tested the hypothesis that *NKRF* is controlled by NF-κB and that the differential activation of distinct NF-κB family members lead to the opposing effect of NE on IL-8/CXCL8 in hASM and lung epithelial cells.

## Results

### NE induces NKRF expression and inhibits IL-8/CXCL8 production in human airway smooth muscle cells but not in A549 cells

**Figure 1:**
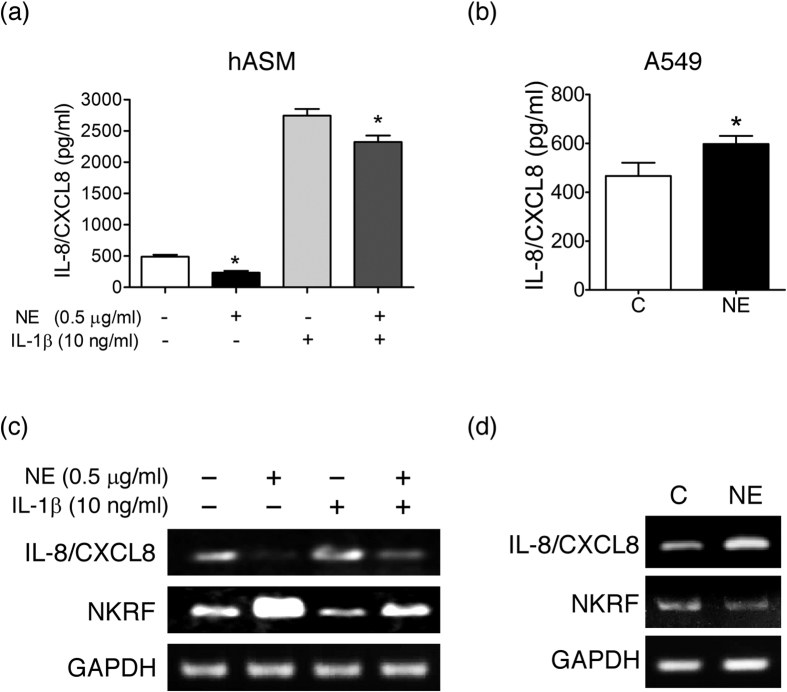
Induction of NKRF and suppression of IL-8/CXCL8 by NE in hASM but not in A549 cells. (**a**,**b**) ELISA of IL-8/CXCL8 release from hASM (**a**) and A549 cells (**b**) stimulated with NE (0.5 μg/ml) in the presence or absence of IL-1β (10 ng/ml) or left unstimulated for 6 h. The results are expressed as the means ± SEM. *p < 0.05 versus the corresponding no NE controls. (C, D) RT-PCR analysis of IL-8/CXCL8 and NKRF mRNA expression in hASM (**c**) and A549 cells (**d**) stimulated with NE (0.5 μg/ml) in the presence or absence of IL-1β (10 ng/ml) or left unstimulated for 2 h. A representative picture of 3 independent experiments with similar results is shown.

Our previous report showed that the incubation of hASM with NE (0.5 μg/ml) for 6 h remarkably suppresses IL-8/CXCL8 release^12^. However, NE stimulates its release from alveolar epithelial A549 cells and bronchial epithelial Beas-2B cells. Similar to our previous report, 0.5 μg/ml NE inhibited IL-8/CXCL8 release in hASM at 6 hours but stimulated its production in A549 cells (Fig. 1a and b). The repressive effect of NE was also observed in IL-1β-stimulated IL-8/CXCL8 in hASM (Fig. 1a). Similar effects were also shown at the mRNA level (Fig. 1c and d). Induction of NKRF specifically occurred in NE-stimulated hASM but not in A549 cells (Fig. 1c and d). We have demonstrated in our previous report that the inducible NKRF causatively mediates the suppressive effect on IL-8/CXCL8 production in hASM^12^.

### NE stimulates distinct NF-κB subunits in hASM and A549 cells

**Figure 2:**
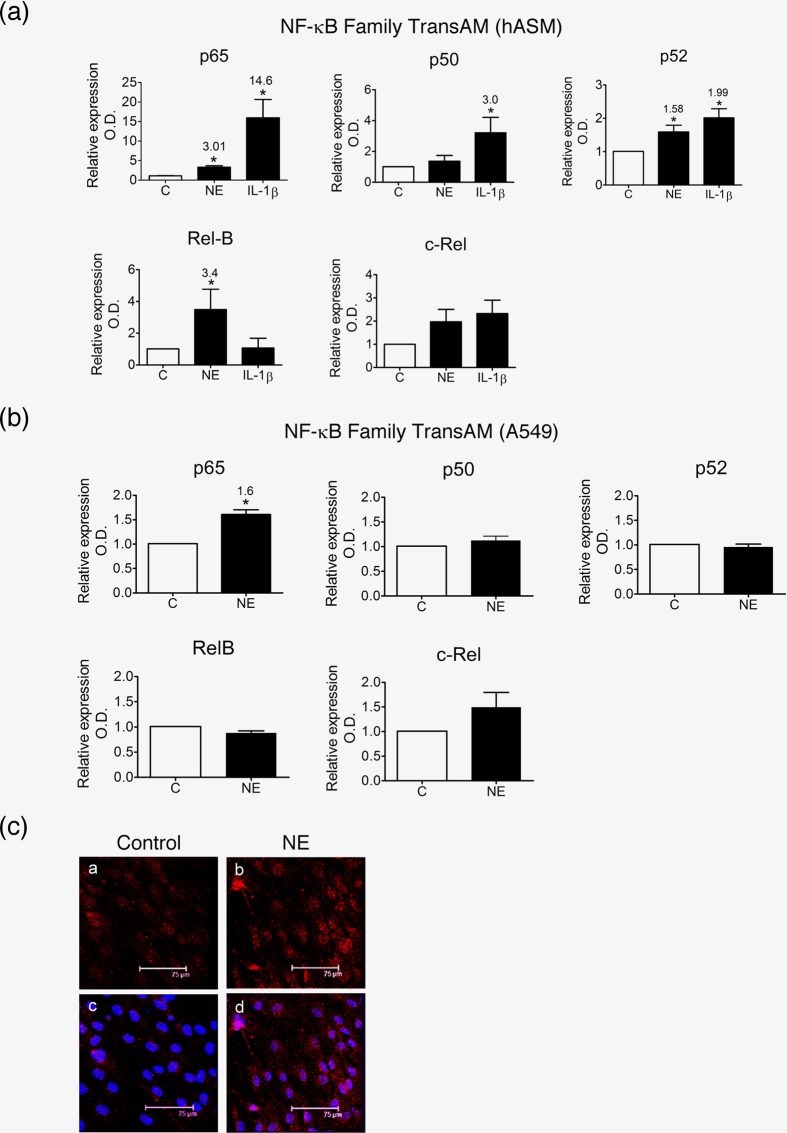
Patterns of NF-κB activation in hASM and A549 cells in response to NE. (**a**,**b**) NF-κB TransAM analysis of hASM (**a**) and A549 cells (**b**) treated with NE (0.5 μg/ml), IL-1β (10 ng/ml) or left untreated for 1 h. The results are expressed as the means ± SEM. *p < 0.05; **p < 0.01 vs. the unstimulated controls. (**c**) Effect of NE on RelB nuclear translocation. Confocal images of untreated and NE-treated hASM cells for 1 h with an antibody specific for RelB (Cy3, **a** and **b**). The images from nuclear staining (Hoechst) were merged (**c** and **d**) to show the subcellular localization of RelB.

NE stimulates NF-κB activation in hASM and A549 cells^12^,^28^. Pretreatment of hASM with the nonspecific NF-κB inhibitor SN50 (10 μM) for 30 minutes abolished the induction of NKRF mRNA (data not shown), suggesting the involvement of NF-κB in NKRF transcription. One possibility for the apparently contradictory roles of NE between hASM and A549 cells is the involvement of different NF-κB subunits in these 2 cell types. To test this possibility, we used NF-κB TransAM, a commercialized NF-κB family DNA binding ELISA. In hASM, NE (0.5 μg/ml) not only stimulated p65 activation by 3.01-fold (p < 0.05) at 1 h but also activated RelB and p52 by 3.4 and 1.58-fold (both p < 0.05), respectively (Fig. 2a). Compared with IL-1β, NE seems to be a weak p65 stimulator; IL-1β stimulated p65 activation by 14.6-fold (p < 0.01) (Fig. 2a). Although IL-1β also stimulated p52 activation, it did not have any effect on RelB. In contrast to hASM, NE only stimulated p65 activation in A549 cells (1.6-fold, p < 0.01) but not other subunits of NF-κB (Fig. 2b). Thus, RelB is specifically activated in NE-stimulated hASM. Activation of RelB was also demonstrated by its nuclear translocation, as examined by confocal microscopic examination of immunofluorescence staining using a specific RelB antibody, (Fig. 2c).

### The induction of NKRF and repression of IL-8/CXCL8 by NE is RelB-dependent

**Figure 3:**
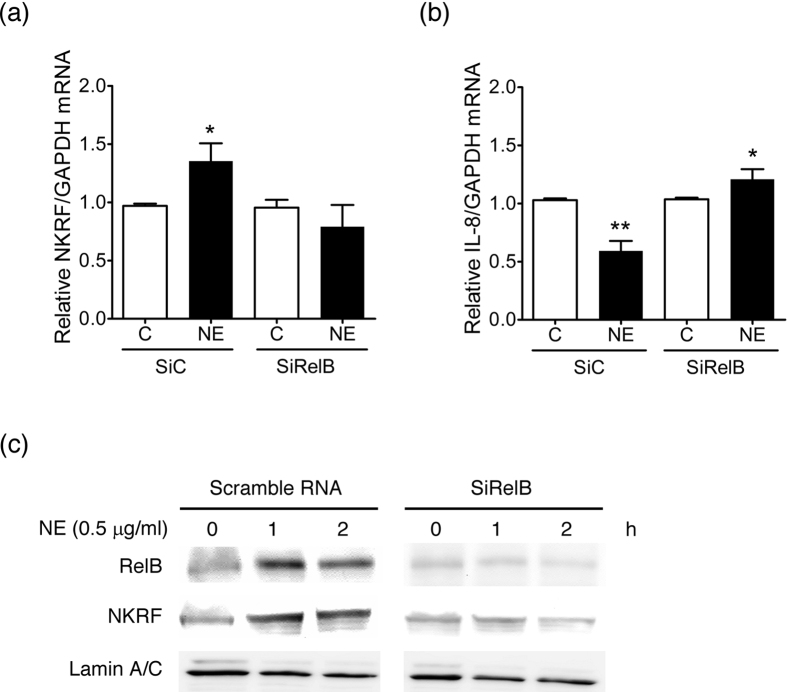
Prevention of NKRF induction and IL-8/CXCL8 suppression in RelB knockdown hASM. (**a**,**b**) Real-time RT-PCR analysis of NKRF (**a**) and L-8/CXCL8 mRNA (**b**) expression in hASM stimulated with NE (0.5 μg/ml) for 2 h in hASM transfected with scrambled RNA (siC) or siRelB. The results are expressed as the means ± SEM. *p < 0.05 versus the corresponding no NE controls. (**c**) Western blot analysis of nuclear RelB and NKRF in scrambled RNA- and siRelB-transfected hASM stimulated with NE (0.5 μg/ml) for 0–2 h. A representative picture of 3 independent experiments with similar results is shown.

To confirm the role of RelB in the induction of NKRF and repression of IL-8/CXCL8, RelB was knockdown by RNA interference (Fig. 3a and b). In contrast to transfection with scrambled RNA (SiC), the knockdown of RelB (SiRelB) prevented the NE-induced expression of NKRF mRNA (Fig. 3a) and protein (Fig. 3c). Furthermore, NE failed to suppress IL-8/CXCL8 expression; instead, it stimulated IL-8/CXCL-8 (Fig. 3b). These data suggest that the NE-induced NKRF and downstream repressive effect on IL-8/CXCL8 is RelB dependent.

**Figure 4:**
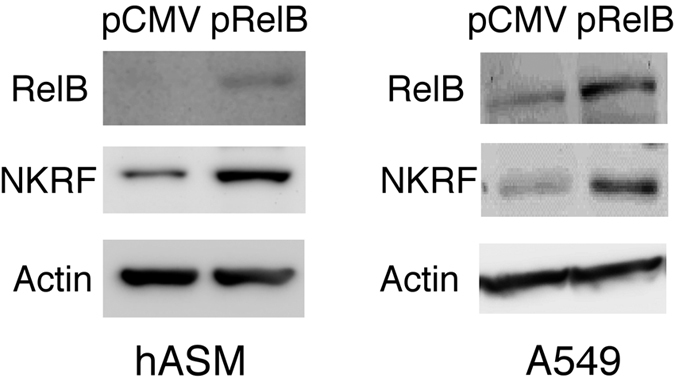
Effect of RelB over-expression on NKRF in hASM and A549 cells. Western blot analysis of RelB and NKRF expression in whole-cell extracts from hASM transected with pRelB or the empty vector pCMV for 24 h. The protein levels of NKRF were increased in hASM- or A549-overexpressing RelB cells compared with the control cells. A representative picture of 3 independent experiments with similar results is shown.

To see whether RelB is adequate for NKRF induction, we performed RelB over-expression experiments by transient transfection with the pCMV-RelB plasmid. Compared with the pCMV empty vector control, the forced expression of RelB induced robust NKRF expression not only in hASM (Fig. 4a) but also in A549 cells (Fig. 4b). Thus, RelB alone can stimulate NKRF expression.

### Recruitment of RelB to the potential κB site in the NKRF promoter

**Figure 5:**
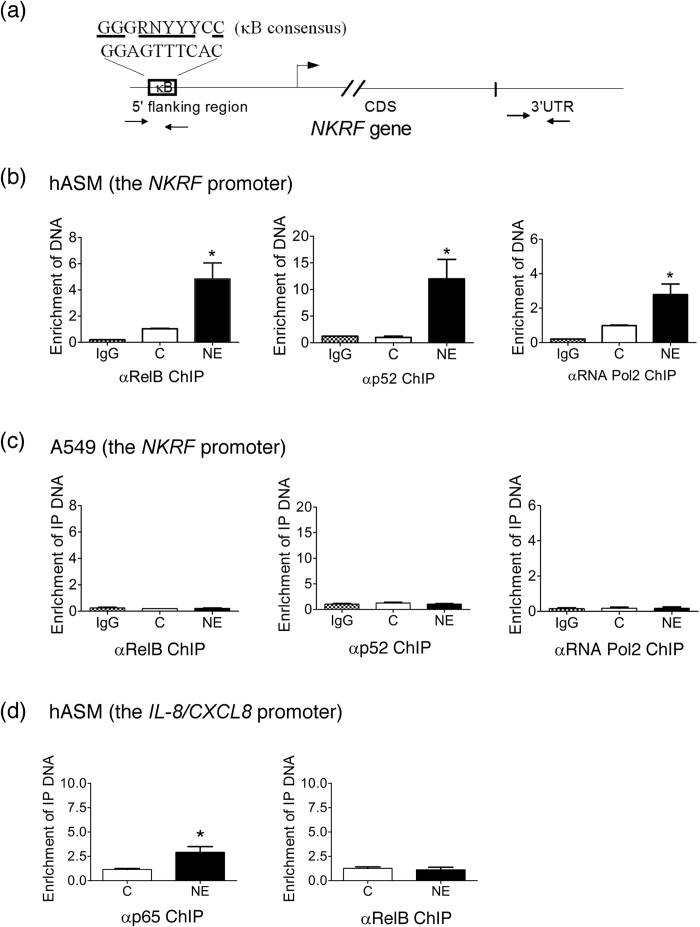
Occupancy of RelB at the *NKRF* promoter. (**a**) Schematic of the *NKRF* promoter. The DNA sequences of the putative NF-κB binding site and κB consensus are indicated. The primer pairs amplifying the κB site and an irrelevant 3′ UTR used in ChIP assays are also indicated. (**b**,**c**) Occupancy of RelB, p52 and RNA Pol2 at the native *NKRF* promoter in hASM (**b**) and A549 cells (**c**). ChIP assay analysis of hASM cells treated with NE (0.5 μg/ml) for 1 h with antibodies specific for RelB, p52 and RNA Pol 2 as indicated. qPCR was used to quantify the IP-DNA. The data are normalized by the input control and are expressed as the means ± SEM relative to the unstimulated control. *p < 0.05 versus the unstimulated control. IgG, preimmune IgG control. (**d**) Occupancy of p65 and RelB at the native *IL*-*8*/*CXCL8* promoter in hASM. ChIP assay analysis of hASM cells treated with NE (0.5 μg/ml) for 1 h with antibodies specific for p65 and RelB as indicated. qPCR was used to quantify the IP-DNA using the primer pairs as previously reported. The data were normalized by the input control and are expressed as the means ± SEM relative to the unstimulated control. *p < 0.05 versus the unstimulated control.

To further confirm the direct role of RelB in the induction of NKRF, ChIP assays were used to observe the protein binding events at the *NKRF* promoter around the potential κB site (Fig. 5a). As shown in Fig. 2, NE mediated RelB, and p52 bound to the NF-κB binding element by NF-κB TransAM analysis. Moreover, the occupancy of RelB and p52 at the NKRF promoter was increased in hASM following NE treatment for 1 h (Fig. 5b). The recruitment of RelB was accompanied by p52, a common partner of the RelB heterodimer. An increased occupancy of RNA polymerase II was also observed, suggesting the transcriptional activation of NKRF promoter via NE stimulation. By contrast, NE did not induce these events around the same site in A549 cells (Fig. 5c).

Our previous report showed that NE mediates NKRF recruitment to the IL-8/CXCL8 promoter and suppression of IL-8/CXCL8 expression in hASM^12^. We next asked whether RelB is bound to the IL-8/CXCL8 promoter. The ChIP assays revealed that RelB was not recruited to this promoter (Fig. 5d). In agreement with general concepts, NE significantly induced the occupancy of p65 at the promoter.

### Induction of the NIK/IKK1/RelB axis by NE in hASM

**Figure 6:**
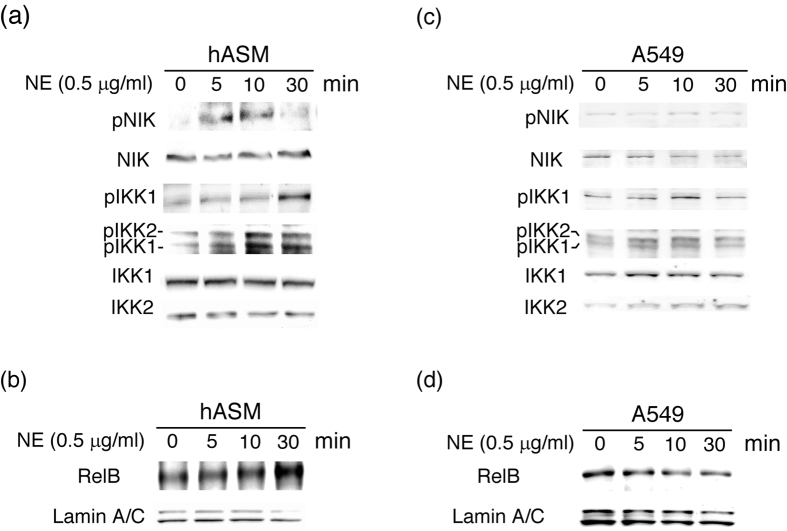
Differential activation of the canonical and non-canonical NF-κB pathways in hASM and A549 cells. Western blot analyses of the upstream kinases of whole-cell extracts (**a**,**b**) and nuclear RelB (**c**,**d**) from hASM (**a**,**c**) and A549 cells (**b**,**d**) treated with NE (0.5 μg/ml) for 0–60 min using specific antibodies for pNIK, NIK, pIKK1, pIKK1/pIKK2, IKK1, IKK2 and RelB as indicated. A representative picture of 3 independent experiments with similar results is shown.

To explore the upstream signals to the differential NF-κB subunits in hASM and A549 cells, time-course studies of potential kinases were conducted using Western blotting. In hASM, NE evoked the rapid and transient activation of NIK at 5–10 min, followed by IKK1 and IKK2 at 10–30 min (Fig. 6). The nuclear translocation of RelB occurred at 30–60 min. In A549 cells, we did not detect consistent activation of NIK and IKK1 during the study period. However, significant but transient activation of IKK2 was observed at 5–10 min. In agreement with the TransAM analysis (Fig. 2c), NE did not activate RelB in this cell line. These data suggest that NIK/IKK1 may be an upstream signal pathway to the non-canonical RelB in hASM.

**Figure 7:**
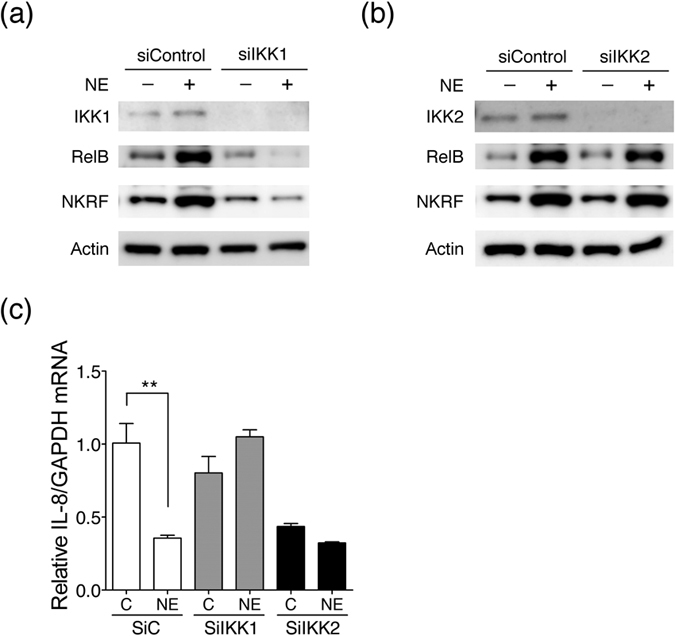
NE mediates the suppression of IL-8 expression by the IKK1-RelB pathway in hASM cells. (**a**) Knockdown of IKK1 in hASM cells, as determined via immunoblotting analysis. IKK1 knockdown abolished the expression levels of RelB after NE (0.5 μg/ml) treatment for 24 hours compared with that in the siRNA control. (**b**) In contrast to IKK1-knockdown cells, NE still mediated the expression of RelB with specific knockdown of IKK2. NE-mediated NKRF expression was reduced in hASM cells with or without NE treatment with specific knockdown of IKK1. (**c**) The mRNA levels of the NKRF downstream target IL-8/CXCL8 were restored in IKK1 knockdown cells after NE treatment. The levels of IL-8/CXCL8 were repressed in hASM IKK2 knockdown cells with or without NE treatment. Moreover, IL-8/CXCL8 levels were also slightly suppressed by NE stimulation with specific knockdown of IKK2.

To further investigate the noncanonical mechanism whereby NE mediates the suppression of IL-8/CXCL8 expression, an approach using specific IKK1 or IKK2 siRNA knockdown in hASM cells was performed. As shown in Fig. 7a, specific knockdown of IKK1 abolished the induction of RelB expression in hASM cells after NE treatment compared with the siRNA control. However, NE also induced the expression of RelB with the knockdown of IKK2 (Fig. 7b). As our expectation, NE-mediated NKRF expression was reduced with the specific knockdown of IKK1 (Fig. 7a). Next, the mRNA levels of the NKRF downstream target IL-8/CXCL8 were rescued in IKK1-knockdown cells after NE treatment (Fig. 7c). By contrast, IL-8/CXCL8 levels were repressed by IKK2 knockdown. Moreover, these results showed that the IL-8/CXCL8 levels were slightly suppressed by NE stimulation with the specific knockdown of IKK2 (Fig. 7c). Our data demonstrated that NE mediated IL-8/CXCL8 suppression in hASM via the NIK/IKK1-RelB signaling pathway.

## Discussion

We had previously reported that NE has differential effects on IL-8/CXCL8 in two major airway structure cells: IL-8/CXCL8 is stimulated in the airway and lung epithelial cells are suppressed in hASM. It turned out that the selectively induced NKRF in hASM played the causative role in the suppressive effect. In this report, we further explored the molecular mechanism underlying the differential effects. We found that NE activated non-canonical NF-κB pathways, e.g., the NIK/IKK1/RelB pathway, transactivate the *NKRF* promoter via a novel κB site in hASM. This pathway was not induced in A549, a lung epithelial cell line and, therefore, has no effect on the *NKRF* gene.

The major finding in the present report is that the *NKRF* is controlled by RelB. Both the Western blotting and TransAM studies showed that NE activated RelB in hASM, the only cellular context with NKRF induction in this study. Using ChIP assays, we could demonstrate that RelB was recruited to the putative κB site in the *NKRF* promoter. Increased occupancy of RNA polymerase II at the same promoter suggests that this recruitment is accompanied by transcription initiation. The function role of RelB in the induction of NKRF was further confirmed by the RelB knockdown experiments. In addition, the forced expression of RelB could induce NKRF, not only in hASM but also in A549 cells. Thus, RelB alone is sufficient for the induction of NKRF. Taken together, RelB is causatively involved in the transactivation of *NKRF* by binding to the *κ*B site in the promoter.

In agreement with previous reports, NE stimulates p65 activation in hASM and bronchial or lung epithelial cells. IL-1β stimulated even more robust activation of p65 in hASM. Intriguingly, in the absence of RelB, activated p65 was not recruited to the κB site in the *NKRF* promoter in NE-stimulated A549 and IL-1β-stimulated hASM. However, we did observe a modest amount of p65 occupancy at this promoter in NE-stimulated hASM, similar to that with RelB. Therefore, it is likely that the κB site in the *NKRF* promoter preferentially recognizes RelB and that p65 might be recruited by its interaction with RelB. More complicated, p65, instead of RelB, was bound to the *IL*-*8*/*CXCL8* promoter in the same NE-stimulated hASM. Therefore, the promoters of the two different genes, *NKRF* and the *IL*-*8*/*CXCL8*, choose distinct NF-κB subunits. This complexity helps sophisticatedly control inflammation in response to environmental stress.

A growing body of evidence has suggested that RelB is implicated in the negative regulation of immune or inflammatory responses. Mice with targeted disruption of RelB developed mixed inflammatory cell infiltration, including neutrophils, in several organs^29^, while RelB-deficient fibroblasts had persistent overexpression of chemokines in response to LPS^30^. It was later reported that RelB is involved in the morphine modulation of proinflammatory and Th1 cytokines^31^ and participates in endotoxin tolerance^24^. In line with these observations, we provided evidence supporting that RelB contributes to the repression of IL-8/CXCL8 in hASM. RelB and p65 have been reported to be mutually antagonistic by forming a transcriptionally inactivate p65/RelB complex, which is unable to bind to κB sites *in vitro*^32^,^33^. As previously discussed, this mechanism does not seem to underlie the NE repressive effect. Therefore, we have discovered a novel mechanism whereby RelB plays a negative regulatory role in inflammation by inducing NKRF. In line with this mechanism, RelB knockdown led to the prevention of IL-8/CXCL8 suppression. Indeed, in the absence of RelB, an A549-mimicked cellular context was created in hASM. We did observe an induction of IL8-CXCL8 in response to NE, instead of repression. Together, the RelB-NKRF axis is a negatively regulatory mechanism for cells to counteract the p65 pro-inflammatory signal.

**Figure 8:**
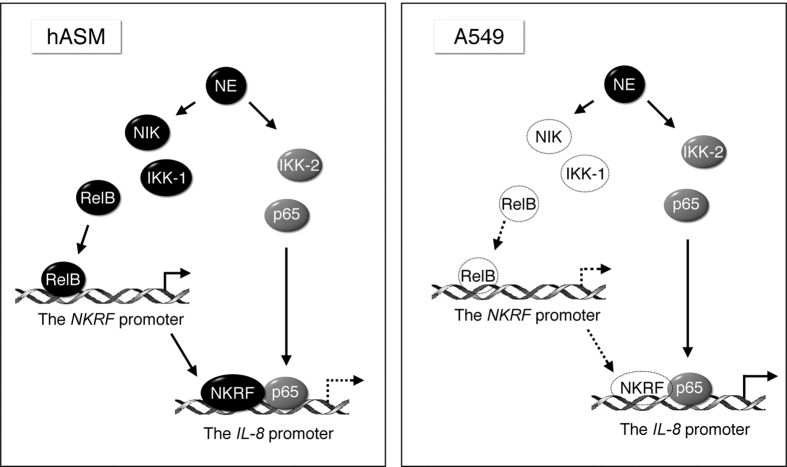
Models for the differential induction of NF-κB and NKRF in response to NE in hASM and A549 cells. In hASM (left panel), NE activates rapidly the non-canonical NIK/IKK1/RelB axis. Nuclear translocated RelB binds to the κB site in the *NKRF* promoter, transactivates and produces NKRF. Although NE stimulates the canonical IKK2/p65 pathway, the inducible NKRF bound at the *IL*-*8*/*CXCL8* promoter abolishes the transcriptional activity of p65 NF-κB, leading to the suppression of IL-8/CXCL8. In A549 cells (right panel), a representative respiratory epithelial cell line, NE only stimulates the canonical NF-κB pathway. In the absence of inducible NKRF, p65 binds to the IL-8/CXCL8 promoter and transactivates it, leading to the enhanced production of IL-8/CXCL8.

Previously, we have reported that NE induces NKRF recruitment to the IL-8/CXCL8 promoter and the removal of RNA Pol II in hASM by ChIP analysis^12^. In this study, we further investigated the NE-mediated induction of NKRF and suppression of IL-8 expression via the noncanonical NF-κB signaling pathway. We found that the upstream signal to RelB is NIK/IKK1. Compared with A549 cells, NE provoked a rapid but transient activation of NIK, followed by IKK1, leading to the nuclear translocation of RelB. TransAM and ChIP assay analysis also demonstrated the co-activation of p52, the common RelB partner. Thus, we identified NE as a stimulator of the non-canonical NF-κB pathway. This NE-mediated effect is cellular context dependent because it is not observed in A549 cells. However, the classical IKK2/p65 NF-κB pathway seems to be more universally activated—both in NE-stimulated hASM and A549 cells. Through this sophisticated mechanism (Fig. 8), NE-mediated responses are tightly controlled in two airway structure cells.

The clinical relevance of NKRF in inflammatory diseases has been rapidly explored as described in the introduction. We have reported that the down regulation of NKRF in COPD patients might be a link to the systemic inflammation of this disease^34^. It is worth studying whether the RelB-NKRF negative regulatory mechanism is dampened in COPD patients and other inflammatory diseases, such as (IBS-D)^7^. In PBMCs from COPD patients, the forced expression of NKRF^12^ could ameliorate the production of inflammatory cytokines. In the present report, the forced expression of RelB could also suppress the production of IL-8/CXCL8. Thus, targeting the NIK/IKK1/RelB/NKRF axis might have therapeutic potential for chronic inflammatory diseases.

## Materials and Methods

### Cell culture and stimulation

Primary hASM was purchased from Cambrex and was prepared according to the manufacturer’s instructions. Cells were grown as described previously^35^. Confluent cells at passages 3 through 7 were starved for 24 h in serum-free medium (SFM) containing Dulbecco’s modified Eagle’s medium (DMEM) supplemented with 4 mM L-glutamine, 2.5 μg/ml amphotericin B, 100 U/ml penicillin, 1% insulin-transferrin-selenium-X, 1:100 nonessential amino acids and 0.1% BSA. After serum deprivation, cells (with or without transfection with siRelB or non-targeting scramble or plasmid, pCMV-XL6 or pCMV-XL-RelB) were incubated for various times (0–24 hours) in fresh SFM containing NE (0.5 μg/ml) or SN50 (10 μM) or IL-1β (10 ng/ml) as indicated in individual experiments.

A549 cells were grown in DMEM containing 10% fetal calf serum (FCS) before incubation for 24 h in SFM. After serum deprivation, cells (with or without transfection of the non-targeting scrambled or expression plasmids, pCMV-XL6 or pCMV-XL-RelB, respectively) were incubated for various times (0–24 h) in SFM-containing NE (0.5 μg/ml), as indicated in individual experiments.

### Reagents

Human NE was purchased from Sigma Chemical Co. (St Louis, MO, USA). The recombinant human IL-1β from Roche Applied Science (Indianapolis, IN, USA). NKRF in whole-cell extracts was detected by Western blotting with a specific antibody as described previously^12^. The antibody specific for human RNA polymerase II (RNA pol 2) used in ChIP assays was from Abcam (Carlsbad, CA, USA) and those for all NF-κB family members were from Santa Cruz Biotechnology Inc. (Santa Cruz, CA). siGENOME SMARTpool targeting human RelB (siRelB) and siCONTROL nontargeting siRelB (siControl) were purchased from Dharmacon (Colorado, USA).

### Enzyme-Linked Immunosorbent Assay (ELISA)

The IL-8/CXCL8 protein concentration in the supernatants from hASM and A549 cells treated with NE (0.5 μg/ml) in the presence or absence of IL-1β (10 ng/ml) were measured using a commercial ELISA kit (R&D Systems, Minneapolis, MN, USA) according to the manufacturer’s instructions.

### Reverse Transcription-Polymerase Chain Reaction (RT-PCR)

Total RNA was isolated from cells using TRIzol reagent (Life Technologies, Rockville, MD) according to the manufacturer’s instructions. The cDNA and PCR protocols were performed described previously^12^.

### Quantitative real-time PCR (qPCR)

qPCR was performed using a LightCycler^®^ 2.0 system (Roche Applied Science) and LightCycler^®^ DNA Master STBR Green I (Roche Applied Science). The PCR protocol was performed described previously^12^. In RT-qPCR, the data were normalized to that of GAPDH. In ChIP assays, the values were normalized to that of the input DNA and were expressed as values relative to the control values. The NKRF promoter:forward:5′-TAT TGA TAT TGG GGA GAT GCC-3′;reverse:5′-GGA TCT TCC TGT CTT TCA TCT-3′. The IL-8/CXCL8 promoter, forward, 5′-GGG CCA TCA GTT GCA AAT-3′;reverse:5′-TTC CTT CCG GTG GTT TCT TC-3′.

### NF-κB DNA binding ELISA

The DNA binding activities of the NF-κB family (p50, p52, p65, c-Rel and RelB) were measured using TransAM NF-κB family kits (Active Motif) according to the manufacturer’s instructions^12^.

### Immunostaining and confocal laser microscopic analysis

Cells were plated onto 24-mm-diameter round coverslips in 6-well plates. After stimulation with NE (0.5 μg/ml) for 1 h, cells were fixed in methanol at −20 °C for 5 min. The cells were then blocked with 1% BSA/PBS at room temperature for 30 min and were incubated with a rabbit anti-human RelB antibody at room temperature for 1 h. Thereafter, the immunostaining and confocal protocols were performed as described previously^12^.

### Western blot analysis

The cytoplasmic and nuclear protein fractions were separated as described previously^36^. The proteins were detected with the specific anti-antibody (NKRF Ab (Kretec);RelB, IKK1 and IKK2 Ab (Upstate); pNIK and NIK Ab (Santa Cruz); pIKK1/2 Ab (Cell signaling) and an alkaline phosphatase-conjugated anti-rabbit secondary Ab (Calbiochem).

### Plasmid construction

The full-length RelB expression vector pRelB was obtained from OriGene Technologies Inc. (Rockville, MD) and was constructed by inserting human RelB cDNA into pCMV6-XL6 vectors (pCMV).

### Transfection of siRNA and plasmids

siRelB, siCONTROL, and plasmid DNA (pCMV and pRelB) were introduced into hASM cells by nucleofection using hASM nucleofector kits, respectively (Amaxa Inc., Gaithersburg, MD, USA), following the manufacturer’s optimized protocols. After nucleofection, the cells were incubated in complete medium for 48 h (for siRelB) or 24 h (for plasmid DNA) before stimulation with NE (0.5 μg/ml) for 6 h (for siRelB).

### Chromatin Immunoprecipitation Assay (ChIP)

ChIP assays were performed as described previously^37^. After stimulation, the protein-DNA complexes were cross-linked at 37 °C for 10 min by formaldehyde (1% final concentration). The protein-bound immunoprecipitated DNA and DNA isolation protocols were performed as described previously^37^. qPCR was performed with 7 μl of DNA sample for quantification. Pre-immune rabbit IgG (IgG control, Santa Cruz) was used to demonstrate the specificity of the antibodies. Primer pairs amplifying an unrelated site in the 3′-UTR of the *NKRF* and *IL*-*8*/*CXCL8* promoters were also used to confirm the site specificity of the assay.

## Additional Information

**How to cite this article:** Ho, S.-C. *et al*. Noncanonical NF-κB mediates the Suppressive Effect of Neutrophil Elastase on IL-8/CXCL8 by Inducing NKRF in Human Airway Smooth Muscle. *Sci. Rep.* **7**, 44930; doi: 10.1038/srep44930 (2017).

**Publisher's note:** Springer Nature remains neutral with regard to jurisdictional claims in published maps and institutional affiliations.
